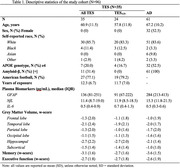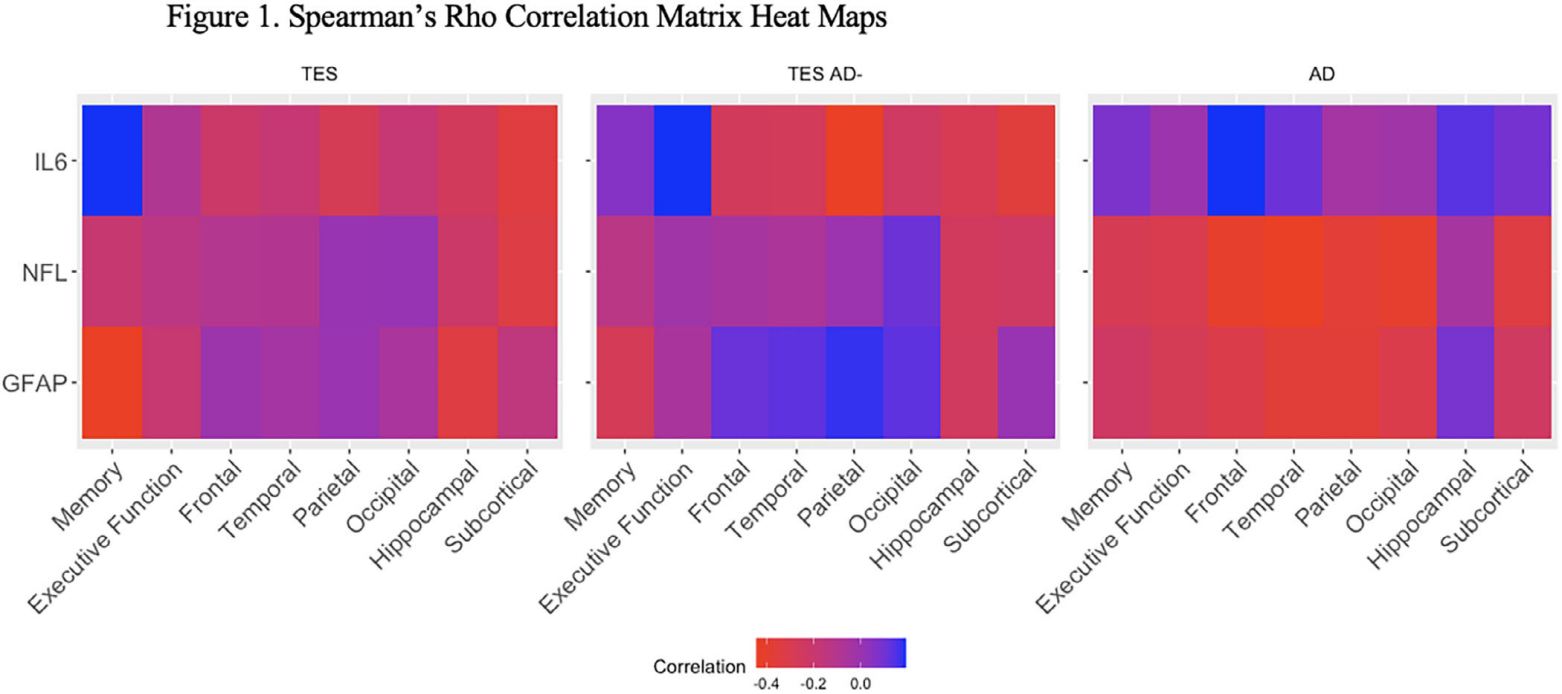# Cognitive and Brain Structure Correlates of Plasma Biomarkers in Traumatic Encephalopathy Syndrome

**DOI:** 10.1002/alz.091768

**Published:** 2025-01-09

**Authors:** Olivia M Emanuel, Emily F Matusz, Shannon Y. Lee, Jessica Bove, Karen Smith, Joel H. Kramer, Gil D. Rabinovici, Breton M. Asken

**Affiliations:** ^1^ University of Florida, Gainesville, FL USA; ^2^ University of California, San Francisco, San Francisco, CA USA; ^3^ University of California San Francisco, San Francisco, CA USA; ^4^ Memory and Aging Center, Weill Institute for Neurosciences, University of California, San Francisco (UCSF), San Francisco, CA USA

## Abstract

**Background:**

Traumatic encephalopathy syndrome (TES) is a proposed framework for the clinical syndrome resulting from chronic traumatic encephalopathy and other neurodegenerative effects of repetitive head impacts (RHI). TES symptoms can mirror Alzheimer's disease (AD) despite absence of hallmark AD pathology. We investigated whether GFAP, NfL, and IL‐6 correlated with cognition and brain volume changes within TES relative to AD.

**Method:**

We studied 96 participants from the UCSF Memory and Aging Center (N=35 RHI/TES; 24 of whom were AD biomarker‐negative, TES_AD‐_; N=61 biomarker‐confirmed AD phenotype, no RHI; Table 1). Plasma was analyzed for GFAP, NfL, and IL‐6 (age‐ and sex‐adjusted). Cognitive measures included composite memory and executive functioning scores (demographically‐adjusted). MRI was obtained concurrent to blood draws and several regions of interest were analyzed (demographic‐ and total intracranial volume‐adjusted): frontal, temporal, parietal, occipital, hippocampus, subcortical. Spearman’s rho assessed associations between plasma biomarkers, cognition, and brain volume. A priori alpha was p<.05, but associations with at least medium effect size (rho ≥0.3) were interpreted as potentially meaningful given small N in TES and TES_AD‐_ subgroups.

**Result:**

In TES, higher GFAP was associated with lower hippocampal volume (rho=‐0.33, p=.09) and worse memory (rho=‐0.44, p=.02). Results were similar in TES_AD‐_ subgroup (GFAP‐hippocampus: rho=‐0.45, p=.07; GFAP‐memory: rho=‐0.42, p=.07). IL‐6 appeared most related to brain volume in the TES_AD‐_ subgroup, where higher IL‐6 correlated with lower volumes in all regions (rho’s ‐.43 ‐ ‐.63, p’s=.004 ‐ .06), but not with cognition. In AD, both higher NfL and GFAP were associated with lower brain volume in several regions and with worse cognition but, notably, neither related to hippocampal volume (NfL: rho=‐.11, p=.47; GFAP: rho=.02, p=.91). IL‐6 did not relate to brain volume or cognition in AD (Figure 1).

**Conclusion:**

Plasma biomarkers reflect different regions of atrophy and cognitive change in TES vs. AD. Inflammation (IL‐6) may play a distinctive role in the pathophysiology underlying atrophy in TES. While recent work highlights astroglial activation (GFAP) is a putative AD‐related biomarker, it may uniquely relate to hippocampal changes in TES. Ongoing work is needed to unravel the clinical utility of plasma biomarkers for characterizing the neurodegenerative effects of RHI and underlying TES symptoms.